# Supplementary material for: P-TRAP: a Panicle Trait Phenotyping tool
Source: BMC Plant Biol. 2013 Aug 29;13:122. doi: 10.1186/1471-2229-13-122 (PMC3848748; doi:10.1186/1471-2229-13-122)
Supplement: Additional file 7 — P-TRAP source code. The Netbeans project that contains the source code of the application. [file 1471-2229-13-122-S7.zip › javadoc.zip!/javadoc/org-bioalg-graphprocessor/org/bioalg/graphprocessor/Path.html]

Path (GraphProcessor)


JavaScript is disabled on your browser.


- Package
- Class
- Tree
- Deprecated
- Index
- Help

*org.bioalg.graphprocessor 1.0*

- Prev Class
- Next Class

- Frames
- No Frames

- All Classes

- Summary:
- Nested |
- Field |
- Constr |
- Method

- Detail:
- Field |
- Constr |
- Method


org.bioalg.graphprocessor

## Class Path

- java.lang.Object
- - org.bioalg.graphprocessor.Path

- ---

    

  ```
  public class Path
  extends java.lang.Object
  ```

  A path is the list of vertices starts and ends by two vertices.

  Author:
  :   faroq

- - ### Constructor Summary

    Constructors

    Constructor and Description

    `Path()`

    `Path(java.util.ArrayList<Vertex> nodes)`
  - ### Method Summary

    Methods

    | Modifier and Type | Method and Description |
    | --- | --- |
    | `void` | `add(int index, Vertex vertex)` |
    | `void` | `add(Vertex vertex)` |
    | `void` | `clear()` |
    | `boolean` | `contains(Vertex vertex)` |
    | `Vertex` | `get(int index)` |
    | `double` | `getlength()` |
    | `java.util.ArrayList<Vertex>` | `getNodes()` |
    | `void` | `setNodes(java.util.ArrayList<Vertex> nodes)` |
    | `int` | `size()` |

    - ### Methods inherited from class java.lang.Object

      `clone, equals, finalize, getClass, hashCode, notify, notifyAll, toString, wait, wait, wait`

- - ### Constructor Detail


    - #### Path

      ```
      public Path()
      ```


    - #### Path

      ```
      public Path(java.util.ArrayList<Vertex> nodes)
      ```
  - ### Method Detail


    - #### getNodes

      ```
      public java.util.ArrayList<Vertex> getNodes()
      ```


    - #### setNodes

      ```
      public void setNodes(java.util.ArrayList<Vertex> nodes)
      ```


    - #### getlength

      ```
      public double getlength()
      ```


    - #### clear

      ```
      public void clear()
      ```


    - #### add

      ```
      public void add(Vertex vertex)
      ```


    - #### add

      ```
      public void add(int index,
             Vertex vertex)
      ```


    - #### contains

      ```
      public boolean contains(Vertex vertex)
      ```


    - #### size

      ```
      public int size()
      ```


    - #### get

      ```
      public Vertex get(int index)
      ```


- Package
- Class
- Tree
- Deprecated
- Index
- Help

*org.bioalg.graphprocessor 1.0*

- Prev Class
- Next Class

- Frames
- No Frames

- All Classes

- Summary:
- Nested |
- Field |
- Constr |
- Method

- Detail:
- Field |
- Constr |
- Method
